# The Impact Mechanism and Spillover Effect of Digital Rural Construction on the Efficiency of Green Transformation for Cultivated Land Use in China

**DOI:** 10.3390/ijerph192316159

**Published:** 2022-12-02

**Authors:** Ying Tang, Menghan Chen

**Affiliations:** School of Public Management, Liaoning University, Shenyang 110136, China

**Keywords:** green transformation, digital village, land use transformation, land use efficiency

## Abstract

Under the context of digital economy, agricultural production will be promoted by implementing the strategy of digital rural construction and giving full play to the role of digital factor productivity. This study systematically explains the mechanism of how digital rural construction affects the efficiency of green transformation for cultivated land use. The panel data of 30 provinces in China from 2011 to 2020 are analyzed through two-way fixed effect, spatial Dubin model and other methods, so as to better understand the impact of digital rural construction on the efficiency of green transformation for cultivated land use and its spillover effect. It is discovered in the study that digital rural construction is effective in enhancing the efficiency of green transformation for regional cultivated land use, and that this promoting effect stands multiple robustness tests. According to the heterogeneity analysis, the promoting effect of digital rural construction is more significant in the eastern region and among the samples with high green transformation efficiency of cultivated land use. In addition to improving the efficiency of green transformation for cultivated land use in the region, digital rural construction can also produce a positive spatial spillover effect to a significant extent. On this basis, the targeted policy recommendations are made in this paper. The first one is to improve the efficiency of green transformation for cultivated land use by accelerating the process of digital rural construction. The second one is to pay close attention to the differences in the process of digital rural construction. The third one is to better understand the “welfare sharing” characteristics of digital rural construction. The last one is to establish a mechanism of regional cooperation.

## 1. Introduction

For a long time, the extensive use of chemical fertilizers, pesticides, herbicides and other chemicals in the process of farmland utilization in China has led to various problems, such as the imbalance of soil nutrients, the decline of fertility and the reduction in organic matter content, which makes soil and water pollution increasingly severe. Meanwhile, it has seriously impaired the supply capacity of high-quality and safe agricultural products in China [1,2]. This poses tough challenges to the protection of cultivated land resources, the sustainable development of agricultural economy as well as the stability of society and the economy [3], which hinders the comprehensive, coordinated and sustainable development of economy and society. It is thus imperative to accelerate the transformation of mode of agricultural development and actively promote the green transformation of cultivated land utilization. Promoting the green transformation of cultivated land utilization is considered an effective strategic solution to resolving the resource and environmental crisis caused by agricultural development [4]. The green transformation of cultivated land utilization can promote the sustainable development of agricultural systems, given the limited water and soil resources carrying capacity, thus maintaining the health of the earth. The green transformation of cultivated land use represents the core idea of green agricultural development. With the promotion of green agricultural development, the complexity of the external environment and the evolution of the food consumption structure, there also new challenges facing the green transformation of cultivated land use [5]. Supported by such basic research as digital earth work and technology support systems, digital technology is applied to construct an information network through software development and hardware integration, which is conducive to agricultural production, management, sustainable development, environmental protection, research and knowledge dissemination [6]. Agricultural technology is expected to assist farmers in making wise decisions, improving crop quality and yield, and reducing the impact of agricultural production on the environment [7]. The digital village is classed as a new variety of information technology, similar to the internet of things, cloud computing, big data and mobile internet, the aim of which is to promote the comprehensive and profound integration of digital and agricultural rural farmers’ production and life [8]. This is beneficial to the improvement of efficiency in the green transformation of cultivated land utilization.

The existing research on the impact of digitization on agricultural and rural areas mainly places focus on the impact of digitization on rural production and life. On the one hand, the impact of digitization on rural life is reflected mainly in the improvement of the cooperative relationship between rural construction subjects, the enhancement of rural governance efficiency [9], the improvement of efficiency in public service supply [10] and the upgrading of farmers’ consumption [11]. On the other hand, the impact of digitization on agricultural production relates mainly to the utility of agricultural digitization. Through data sharing, digital agriculture empowers farmers to make wise decisions, significantly improve labor productivity and crop yield, reduce energy and material costs and mitigate the impact on the environment [12]. In rural areas with low levels of digital infrastructure, it makes up for other facilities [13]. With the support of basic digital earth work and technology support systems, digital agriculture builds an information network through software development and hardware integration, for practical application in various fields such as detection, transmission and storage of information, artificial intelligence and cloud technology, as well as the management decision-making based on robot technology [14]. This plays a positive role in the optimization of agricultural production process [15], the improvement of management systems [16] and the expansion of product sales channels [17], environmental protection [12], sustainable development and knowledge dissemination. With digital technology adopted to monitor the use of agricultural subsidies, the efficiency of fund use can be improved and government officials can be prevented from abusing their power and misappropriating agricultural subsidies [18]. The practice of digital agriculture contributes to the rapid development of agriculture in various countries [6], which is a positive response from the relevant application subjects [19]. Gaining a better understanding of land use and soil conditions can promote the development of agricultural production [20]. The application of digital technology can make users fully understand the impact of different use methods on ecosystem services, with useful information provided for better decision-making on the improvement of land management and ecosystem service quality [21].

Digitization facilitates the transformation of cultivated land use by improving the management system of cultivated land use [22], optimizing crop rotation [23], estimating agricultural irrigation water consumption [24] and improving the operability of agricultural machinery [25]. The impact of digitization on cultivated land use is reflected mainly in the use of digital soil maps, digital elevation models, satellite images and climate data, along with the application of remote sensing and geographic information system technologies to analyze the characteristics of soil quality [26,27], monitor the changes in cultivated land [28] and environmental changes [29], as well as analyze and predict the impact of changes in cultivated land use on natural landscapes [30]. This not only provides technical support for preserving biodiversity, maintaining and strengthening ecosystem services [31,32], but also creates opportunities for the green use of cultivated land. The green transformation of cultivated land use represents a new trend under the context of agricultural green transformation. Prompted by the trend of green agricultural development, scholars have gradually paid attention to the adverse impact of cultivated land use on the environment [33], the sustainability and stability of existing cultivated land use models, and other issues. Manifested as the temporal change of regional cultivated land green use patterns, the green transformation of cultivated land use is the constant improvement and innovation of cultivated land use system elements with green development as the premise, to guide cultivated land use activities towards green development [34]. Emphasizing the trinity of “quantity, quality and ecology” [35], the green transformation of cultivated land use aims to achieve the dual goals of “green” and “transformation”. This provides an effective solution to achieving the coordinated development of cultivated land use and the environment [36].

As for the existing studies on the transformation of cultivated land use, most of them focus on the theory and research framework of the transformation of cultivated land use [37], the transformation form of cultivated land use [38], the spatiotemporal evolution characteristics and mechanism [39], as well as the transformation influencing factors [40], with the emphasis placed more on the spatial transformation and functional transformation of cultivated land use [41]. Despite some studies paying attention to the impact of cultivated land use on green development, there are still few studies focusing on the green use of cultivated land in combination with rural digitization. Therefore, this study starts with the mechanism of how rural digitization affects the green transformation of cultivated land use. Then, a discussion is conducted about the impact of rural digitization on the efficiency of green transformation for cultivated land use from three perspectives: the motivation of farmers, the opportunity to choose green transformation of cultivated land use and the ability to do so. The proposed hypotheses are tested mathematically to provide theoretical reference for improving the efficiency of green transformation of cultivated land use. The contributions of this study are detailed as follows. Firstly, the development effect of green use of cultivated land is accurately reflected by giving careful consideration to the negative effects of carbon emissions and non-point source pollution caused by cultivated land use. Secondly, some research assumptions are made under a research framework on the motivation, opportunity and ability of rural digital construction to affect the adoption of green transformation behavior of cultivated land use by farmers. Furthermore, China’s provincial panel data are used to empirically explore the impact of rural digital construction on the efficiency of green transformation of cultivated land use. Lastly, by further analyzing the spatial factors, this study explores the spatial spillover effect of rural digital construction on the efficiency of green transformation for regional cultivated land use. This enriches the research on the efficiency of rural digital construction and regional cultivated land use green transformation from a spatial perspective.

## 2. Theoretical Analysis and Research Hypothesis

### 2.1. Impact Mechanism Analysis

As a classic theory in behavioral organization theory, motivation opportunity ability (MOA) theory [42] is proposed to explore the influencing factors for individual decision-making. According to this theory, the decision-making of individual behaviors is influenced by the three factors: motivation, opportunity and ability. Among them, motivation refers to the driving force that motivates individuals to engage in certain activities. Opportunities refer to the favorable conditions faced by individuals when they engage in certain activities, such as advanced technology and equipment, favorable market environment and high financial subsidies. Ability refers to the internal conditions required by individuals to implement decision-making behaviors, such as knowledge reserves and material capital, etc. Whether the production behavior of farmers, who are the direct decision-maker and executor of cultivated land use, can be “green” is essential for improving the efficiency of green transformation of cultivated land use. Therefore, the MOA theory is applied in this section to construct a framework for analyzing the impact of digital rural construction on the efficiency of green transformation for cultivated land use.

Firstly, the construction of the digital countryside can support the efforts to cultivate the awareness and concept of green production among farmers, thus improving the efficiency of green transformation of cultivated land use on motivation levels. There are three factors that affect the green transformation of cultivated land utilization. One is the innovation of agricultural technology and another is the upgrading of production equipment. The last one is the transformation of farmers’ understanding about the relationship between agricultural production and the ecological environment [43]. Therefore, the concept of green development is crucial to the whole process of cultivated land use, so as to promote the shift from the traditional extensive cultivated land use mode characterized by “high input, high output and high pollution” [44] to the green cultivated land use mode which is characterized by “resource conservation and environmental friendliness”. Digital technology can provide farmers with diversified information, help them eradicate backward thinking and enhance the recognition given to the green transformation of cultivated land [45]. In addition, the main body of digital information dissemination tends to attract the attention of netizens through negative news coverage. Compared with positive news, negative news is more likely to trigger emotional resonance among netizens, cause tension and anxiety and prompt netizens into riskier avoidance behaviors [46]. When farmers are exposed to a lot of negative news about farmland pollution, food safety and other issues, they will re-examine whether their mode of agricultural production is reasonable. The formation of awareness and concept of green production among farmers will translate into conscious behavior, which motivates farmers to engage in the green transformation of cultivated land use, thus improving the efficiency of green transformation for cultivated land use.

Secondly, the construction of digital countryside is conducive to improving the technical support for agricultural production and enhancing the efficiency of green transformation for cultivated land utilization from the perspective of opportunity. The efficiency of cultivated land utilization is affected not only by natural factors such as landform, soil fertility, climate and hydrology, but also by various social and economic factors such as production technology and infrastructure level. Natural factors play an increasingly important role in the practice of modern agricultural production [47]. Digital rural development is beneficial to integrate modern production technology and infrastructure into the utilization of cultivated land resources, upgrade the utilization mode of cultivated land [48] and enhance the efficiency of green transformation for cultivated land utilization through technological innovation. Specifically, through the agricultural big data system, farmers can take advantage of the sensor nodes and information transmission network of agricultural production sites, satellite remote sensing, unmanned aerial vehicles and other tools to collect the data and images related to agricultural product planting conditions, growth status, natural disaster prediction and other data. Remote real-time monitoring and tracking are performed through the use of smart phones, computers and other devices for the intelligent perception of agricultural production, which is required to formulate targeted production decision-making schemes (such as appropriate input of chemical fertilizers, pesticides, etc.). On the one hand, intelligent and precise agricultural production is conducive to lowering factor input costs and improving the efficiency of agricultural production and resource utilization. On the other hand, the input of agricultural factors, especially such agricultural chemicals as chemical fertilizers and pesticides, is a major contributor to agricultural carbon emissions [49] and environmental pollution [50]. Intelligent and precise agricultural production is effective in reducing the amount of chemicals used in the process of farmland utilization, achieving clean production and waste recycling, reducing the negative externalities of agricultural production, and improving the efficiency of green transformation for farmland utilization.

Finally, the construction of digital countryside supports farmers in increasing their material capital and improving the efficiency of green transformation for cultivated land use on the capacity level. As economic people, farmers have to make rational choices on the adoption of green transformation behavior and the degree of transformation after carefully weighing up their own existing material capital, which is stimulated by the change of green development awareness and the innovation of production technology conditions. The efficiency of green transformation for cultivated land use may be constrained by the fact that the material capital possessed by farmers cannot meet the needs of corresponding behaviors, as a result of which they show a low level of willingness or exclusion [4]. There are two ways that digital rural construction affects the material capital level of farmers: the increase of financing capacity and the improvement of incomes. On the one hand, the construction of digital countryside enables farmers to better understand and identify other financing channels through the internet and other ways to improve financing efficiency [51]. With the updating of rural information technology, digital inclusive finance gains popularity in rural areas. By reducing the cost of services offered by traditional financial institutions, it improves the permeability of financial services and expands the coverage of financial services. It provides more efficient channels of financing for the vulnerable groups in rural areas who have been excluded from the financial market for a long time [52]. On the other hand, digital rural construction can not only promote the integration and upgrading of rural primary, secondary and tertiary industries, but also increase the income earned by farmers. The construction of the digital countryside has given rise to many emerging economic forms. For example, the construction of e-commerce platforms is promoted in some rural areas to expand the channels of agricultural product sales, promote the integration and development of traditional agriculture and manufacturing, service industry, logistics and other industries, and facilitate the vertical extension of the agricultural industry chain [53]. Through the clustering effect and scale effect of industrial cooperation under the context of high-level rural digitization, farmers have their income boosted. In addition, with the rise of the internet economy, farmers can rely on the internet to develop sidelines, which improves their incomes, enables them to engage in the green transformation of cultivated land use and thus enhances the efficiency of green transformation for cultivated land use. Based on the above analysis, the following hypothesis is proposed:

**H1.**  *Digital rural construction is conducive to improving the efficiency of green transformation for cultivated land utilization.*

### 2.2. Spatial Spillover Effect

Furthermore, while promoting the efficiency of green transformation for cultivated land use in this region, digital rural construction can also produce a positive spillover effect on the efficiency of green transformation for cultivated land use in the surrounding areas. It is mainly reflected in the fact that the widespread application of digital technology in the context of digital rural construction accelerates the transmission of information between different regions. The increasing width and depth of interregional economic activities, production and life enable knowledge, technology, systems and policies to break the constraints of time and region, thus promoting the rapid flow and renewal of production factors. In this context, the in-depth interaction and exchange between regions can be achieved to promote the process of knowledge, technology, systems and policies. On the one hand, it can optimize the production concept and production capacity of farmers, thus improving the efficiency of green transformation for cultivated land use. On the other hand, due to the demonstration effect, the regions with relatively insufficient systems and policies follow suit in adopting the systems and policies related to the use of cultivated land in the adjacent high efficiency areas to reduce the cost of system innovation. Based on the above analysis, the following hypothesis is proposed:

**H2.**  *Digital rural construction has a positive spillover effect on the efficiency of green transformation for cultivated land use.*

## 3. Materials and Methods

### 3.1. Analytical Methods

In order to test the overall impact of digital rural construction on the efficiency of green transformation for cultivated land use, the following benchmark regression model is constructed:

(1) $${\mathrm{GTCLU}}_{\mathrm{it}}{=\beta }_{0}{+\beta }_{1}{\mathrm{Dig}}_{\mathrm{it}}{+\beta }_{2}{\mathrm{Controls}}_{\mathrm{it}}{+\varepsilon }_{\mathrm{it}}$$

where GTCLU represents the efficiency of green transformation for cultivated l and use; Dig indicates the level of digital rural construction; ε denotes a random disturbance term; i refers to the province; and t represents the year.

From the spatial perspective, a spatial econometric model is further constructed to explore the spatial spillover effect of digital rural construction on the efficiency of green transformation for cultivated land utilization. The commonly used spatial econometric models include the spatial error model (SEM), spatial autoregressive model (SAR) and spatial Dubin model (SDM). The spatial Dubin model (SDM) is divided into endogenous and exogenous interaction effect models. With careful consideration given to the spatial correlation of the missing variables that have an impact on the dependent variables to some extent, it improves the accuracy of results of empirical analysis, which makes it superior to other models [54]. Therefore, the spatial Dubin model (SDM) is adopted in this study to test the spatial spillover effect. The model is expressed as follows:

(2) $$\begin{array}{cc} {\mathrm{GTCLU}}_{\mathrm{it}} & =\beta_{0}+\partial \sum_{j}w_{\mathrm{ij}}{\mathrm{GTCLU}}_{\mathrm{jt}}+\beta_{1}{\mathrm{Dig}}_{\mathrm{it}}+\tau_{1}\sum_{j}w_{\mathrm{ij}}{\mathrm{Dig}}_{\mathrm{jt}}+\beta_{2}{\mathrm{zControls}}_{\mathrm{it}}+\\ \; & \tau_{2}\sum_{j}w_{\mathrm{ij}}{\mathrm{Controls}}_{\mathrm{jt}}+\mu_{i}+\gamma_{t}+\varepsilon_{\mathrm{it}} \end{array}$$

where $w_{\mathrm{ij}}$ represents the spatial weight matrix, or the binary adjacency matrix used in this study to be specific; $\mu_{i}$ indicates the fixed effect of the province; $\gamma_{t}$ denotes the fixed effect of the year. Other variables have the same meanings as Equation (1).

### 3.2. Variables and Indicator System

(1) Explained variable: the green transformation efficiency of cultivated land use (GTCLU). Based on the connotation of green transformation of cultivated land use and existing research results [47,55,56,57], an input and output evaluation index system is established in this study (Table 1) that involves a composite system of “resources, economy, society and ecological environment” to measure GTCLU. Among them, the input indicators include land, labor, technology and capital, to reflect the green “factor transformation” in the green transformation development of cultivated land utilization. The land input adopts the indicator of the sown area of crops per worker, to reflect the level of large-scale land management in the process of green transformation of cultivated land use. The labor input involves two indicators: the number of employees in the planting industry and the number of agricultural technicians in state-owned enterprises and institutions. The former reflects the input level of general cultivated land labor force, while the latter reflects the input level of high-quality agricultural talents in the green transformation of cultivated land utilization. As for the technical input, it is represented by the comprehensive utilization rate of agricultural machinery in the process of crop farming and harvesting, to reflect the level of mechanization in the process of farming, planting, harvesting and other links of production during the green transformation of cultivated land utilization. The capital input is represented by the actual use of physical capital commonly used in the process of farmland utilization, including the amount of chemical fertilizer converted, the total power of agricultural machinery, the use of pesticides, the use of agricultural film, the area of irrigation input and the use of diesel.

The output indicators include expected output and unexpected output. The former includes two elements. One is the economic benefits created by the cultivated land use system given a certain level of factor input, which is expressed as the output value of the planting industry. The other is the grain supply provided by the cultivated land utilization system to ensure the national food security, which is expressed as grain output. Two unexpected output indicators, agricultural carbon emissions and agricultural non-point source pollution, are used to reflect the negative effects for the ecological environment in the process of cultivated land use. Among them, agricultural carbon emissions are the carbon emissions from five sources during the process of farmland utilization: chemical fertilizer, pesticide, agricultural film, irrigation facilities and agricultural diesel input. Based on reference [58], the IPCC carbon emission coefficient method is adopted in this study. The emission coefficient of the above carbon emission sources is detailed as follows. It is 0.8956 (kg/kg) for chemical fertilizer, 4.3941 (kg/kg) for pesticide, 5.18 (kg/kg) for agricultural film, 0.18 (kg/kW) for agricultural machinery and 25 (kg/hm^2^) for irrigation. Agricultural non-point source pollution mainly refers to the non-point source pollution emissions caused during the use of cultivated land, as manifested in the excessive use and residual pollution of various agricultural chemicals such as chemical fertilizers, pesticides and agricultural film, etc. In the present study, the amount of nitrogen (phosphorus), pesticide and agricultural film loss is used to characterize the emissions of pollutants from the use of cultivated land. For the relevant loss coefficients, they are obtained from the manual on agricultural pollution source coefficients published by the National Pollution Source Census of China, with consideration given to the impact of regional differences as much as possible for estimation [59].

To select the measurement methods suitable for the green transformation efficiency of cultivated land use, scholars mainly rely on data envelopment analysis (DEA) and stochastic frontier analysis (SFA). The DEA method is advantageous in processing data with multiple inputs and outputs, and it removes the need to set specific production function forms, thus reducing the subjective controversy of research. For this reason, it has become a popular choice for scholars both at home and abroad. However, there are some problems with the traditional DEA model, such as the relaxation of input and output and the inability to fully consider the unexpected output factors. Therefore, the super-efficiency SBM model was proposed by Tone in 2003. This model not only solves the relaxation of input and output, but also takes into account the unexpected output [60]. The model is expressed as follows:

(3) $$\begin{array}{c} \rho =\min\frac{1-\frac{1}{N}\sum_{n=1}^{N}\left(S_{n}^{x}/x_{k^{\prime }n}^{\prime }\right)}{1-\frac{1}{M+1}\left\{\frac{\sum_{m=1}^{M}S_{m}^{y}}{y_{k^{\prime }m}^{t^{\prime }}}+\sum_{i=1}^{l}S_{i}^{b}/b_{k^{\prime }i}^{t^{\prime }}\right\}}\\ \;s.t.\;\sum_{i=1}^{T}\sum_{k=1}^{K}z_{k}^{t}x_{kn}^{t}+S_{m}^{y}=x_{k^{\prime }n^{\prime }}^{t^{\prime }}\left(n=1,\cdots ,N\right)\\ \sum_{t=1}^{T}\sum_{k=1}^{K}z_{k}^{t}x_{km}^{t}-S_{m}^{y}=y_{k^{\prime }n^{\prime }}^{t^{\prime }},\left(m=1,\cdots ,N\right)\\ \sum_{i=1}^{T}\sum_{k=1}^{K}z_{k}^{t}b_{ki}^{t}+S_{i}^{b}=b_{k^{\prime }i^{\prime }}^{t^{\prime }}\left(i=1,\cdots ,N\right)\\ z_{k}^{t}⩾0;S_{n}^{x}⩾0;S_{m}^{y}⩾0;S_{i}^{b}⩾0,\\ \left(k=1,\cdots ,K\right) \end{array}$$

where ρ represents the economic efficiency value of the evaluation unit; the evaluation unit consists of input *N*, expected output *M* and unexpected output *I*; *n*, *m* and *i* represent the type of indicators of investment, expected and unexpected output, respectively; *T* refers to time; *x*, *y* and *b* denote the type of loose variables; $S_{n}^{x}$, $S_{m}^{y}$ and $S_{i}^{b}$ represent the loose variables of input, expected output and unexpected output, respectively; $x_{k^{\prime }n}^{t^{\prime }}$, $y_{k^{\prime }n}^{t^{\prime }}$ and $b_{k^{\prime }i}^{t^{\prime }}$ indicate the input-output value at $t^{\prime }$ prime time on the $k^{\prime }$ prime unit, respectively; and $z_{k}^{t}$ refers to the weight of the evaluation unit.

(2) Core explanatory variables: digital rural construction level (Dig). The impact of digital rural construction on agricultural production is manifested mainly in the dissemination and sharing of information, digital production and service application. Based on literature [61,62] and the availability of data, the evaluation index system of digital rural construction level is constructed in this study from two perspectives: the level of digital rural infrastructure construction and the level of digital rural application service. Among them, digital rural infrastructure is a precondition to the development of digital countryside. In the digital era, the development of information infrastructure (premised on the new generation of information technology represented by the Internet, and the integrated infrastructure developed by the digital and intelligent transformation of traditional infrastructure using the new-generation information technology) can provide the crucial support for rural digital transformation. Therefore, it is measured by the average number of computers per 100 rural households, the average number of mobile phones per 100 households and the number of rural broadband access users. The level of service related to digital village application is represented by the deepening development of digital village construction. Reflecting the digital development of rural production and life, it is measured by the total index of digital inclusive finance and the length of rural delivery route. The weight of the evaluation index system is determined by using the entropy method. The evaluation index system and the weight of the indexes are shown in Table 2. It can be seen from the table that the weight of the number of rural broadband access users is the largest, indicating that broadband networks have become the most significant driving force for digital rural construction. At the same time, the weight of the average number of mobile phones owned by rural households per 100 households is the least significant. The possible reason for this is that the rate of mobile phone penetration is generally high in China, which limits its contribution to digital rural construction.
(3) Control variables. In order to apply control on the impact of other factors on the green transformation efficiency of cultivated land use, the following indicators are treated as control variables in this study. The first one is disaster-affected area (Dis), which is measured by the sum of disaster-affected area and disaster-affected area of crops. The second one is the rural medical facility level (Med) as measured by the number of village clinics per 10,000 people in rural areas. The third one is measured by the ratio of grain sown area to total crop sown area. The last one is the multiple cropping index (Multi) as measured by the ratio of the total sown area of crops to the cultivated area.

### 3.3. Data Resources

In the present study, the panel data of 30 provinces in China (excluding Tibet, Hong Kong, Macao and Taiwan) from 2011 to 2020 are selected as the sample data. The input and output data of cultivated land use are sourced from the “China Statistical Yearbook”, “China Rural Statistical Yearbook”, “China Agricultural Machinery Industry Yearbook” and “China Science and Technology Statistical Yearbook”. The data on the level of digital rural construction are collected from the “China Statistical Yearbook” and the report on digital inclusive finance index as compiled by the Digital Finance Research Center of Peking University. Table 3 shows the descriptive statistics of each variable.

## 4. Analysis of Empirical Results

### 4.1. Benchmark Regression Analysis

Table 4 lists the benchmark regression results of digital village construction (Dig) on green transformation efficiency of cultivated land use (GTCLU). POLS refers to a mixed OLS model. The results show that the estimation coefficient of digital rural construction is significantly positive at the 1% statistical level. OLS-FE1 indicates the fixed effect model. Before that, the *p* value was 0.000 as calculated by the Hausman test, which supports the use of the fixed effect model for parameter estimation. After control is applied on the fixed effect of provinces and years, the estimated coefficient remains significantly positive at the 1% statistical level. OLS-FE2 shows the estimated result as obtained after the introduction of relevant control variables into the fixed effect model, and the conclusion remains unchanged. It suggests that digital rural construction is conducive to improving the efficiency of green transformation for cultivated land use. From the perspective of control variables, the estimated coefficient of disaster affected area (Dis) is significantly positive at the 1% statistical level. The possible reason for this is that the disaster suffered by crops reduces the output of agricultural products, which is adverse to improving the green transformation efficiency of cultivated land utilization. The estimated coefficient of rural medical facility level (Med) is found significantly positive at the 5% statistical level. This is because medical facilities are the typical public goods that can be used to improve the efficiency of agricultural production by ensuring the health of agricultural labor. The estimation coefficient of the multiple cropping index (Multi) is also found to be significantly positive at the 1% statistical level. The possible reason for this is that the increase of multiple cropping index means the rising intensity of cultivated land use, which may increase the output, thereby improving the efficiency of green transformation of cultivated land use. The estimation coefficient of plant structure fails the significance test.

### 4.2. Robustness Test

(1) Replace the model. Considering that all the values of cultivated land use green transformation efficiency (dependent variable) measured in this study reach above 0, which conforms to the conditions required for the restricted dependent variable model, the Tobit model of the restricted variable model is applied for re-estimation. By comparing the estimated results of Tobit model and benchmark model, it can be found out that there is no change to the direction of influence exerted by the estimated coefficient of digital rural construction level, and that the estimated results are consistent with the conclusions of benchmark results after the model is replaced.
(2) The samples collected from municipalities are excluded. Considering that the agricultural production and operation activities of municipalities directly under the Central Government are significantly different from those of other provinces, the sample data of Beijing, Tianjin, Shanghai and Chongqing are further excluded from this study, with the remaining (260-count) sample data used for re-estimation. After the introduction of control variables and adopting fixed effects, the estimated coefficient of digital rural construction level remains significantly positive at the 1% statistical level, and the research conclusion remains valid.
(3) Lagging independent variable. Since the digital rural construction may show a lag effect, lagging one period of the digital rural construction level (L. Dig) as a new independent variable are re-estimated. After the introduction of control variables and considering the fixed effect, the estimated coefficient of the level of digital rural construction remains significantly positive, indicating the “snowball effect” of digital rural construction. That is to say, the current digital rural construction contributes to improving the green transformation efficiency of cultivated land utilization in the next phase.
(4) Endogenous treatment. There are two reasons for the endogenous problem encountered in this study. On the one hand, despite the control applied on several variables for modeling, there remain some missing variables, which leads to the deviation in the regression results. On the other hand, although the digital rural construction has a promoting effect on the green transformation efficiency of cultivated land use, the demand for digital rural construction also increases with the improvement of efficiency in the green transformation of cultivated land use. That is to say, a two-way causal relationship may exist between the two. Therefore, the instrumental variable method is adopted in the present study to reduce endogenous problems. When the tool variable method is used to make an estimation, it is necessary to select the appropriate tool variables. In this study, the number of postal service outlets per million people in rural areas is used and the natural logarithm (ln. Post) is taken as the instrumental variable [63]. This is because digital information technology is the continuation of traditional communication technology, and the distribution of traditional postal services affects the early development of digital rural construction, which meets the requirements on the “relevance” of tool variables. Furthermore, the frequency of applying traditional information tools represented by postal services declines gradually, and it has little impact on cultivated land use, thus meeting the “exogenous” requirements of tool variables. Table 5 shows the results of estimation based on instrumental variable method. The *p* value of endogenous test is 0.004, which rejects the hypothesis that there is no endogenous problem. That is to say, there is an endogenous problem in the model. The result of the weak identification F test shows that the tool variable rejects the hypothesis at the level of 1%. That is to say, there is no weak tool variable problem. The test results demonstrate the estimation results as reliable and the research conclusions as valid. Thus, the hypothesis H1 proposed in this study is supported.

### 4.3. Heterogeneity Analysis

**Figure 1:**
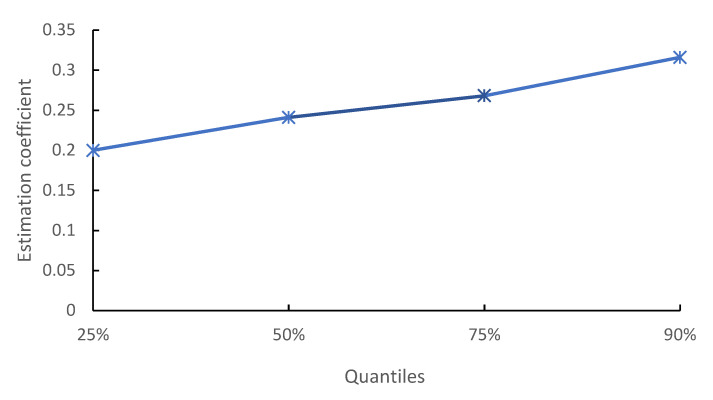
Quantile feature.

(1) Regional heterogeneity. Considering the different geographical conditions, there may be regional heterogeneity in the impact of digital rural construction level on the green transformation efficiency of cultivated land use. According to the distribution characteristics of China’s geographical location, the sample data can be divided into three regions: the east, the middle and the west. Therefore, the fixed effect model is used to test regional heterogeneity, and the regression results by region are shown in Table 6. According to the results, the estimated coefficients of the digital rural construction level in the three regions are significantly positive, indicating that the improvement of the digital rural construction level is accompanied by a significant improvement to the green transformation efficiency of cultivated land use in each region. In addition, the estimated coefficient is higher in the eastern region than in the central and western regions, because the higher economic level of the eastern region is conducive to digital rural construction and can enhance regional advantages.
(2) Heterogeneity of distribution. In addition, panel unconditional quantile regression model is used in this study to explore the impact of digital rural construction level on the green transformation efficiency of cultivated land use at different quantile levels. Table 7 and Figure 1 show the estimated coefficients of the impact of digital rural construction level on the green transformation efficiency of cultivated land use at the quantile of 25%, 50%, 75% and 90%, respectively. The estimation coefficients at each quantile are positive, passing the 1% significance level test. However, considering the size of the estimation coefficient, the improvement of the quantile level is coupled with a gradual increase in the estimation coefficient of the digital village construction level on the green transformation efficiency of the cultivated land use. It indicates that the promotion of the digital village shows an increasing trend of marginal utility.

## 5. Discussion

### 5.1. Spatial Autocorrelation Test

The results of the global spatial autocorrelation test are shown in Table 8. It can be seen from the table that the global spatial autocorrelation coefficient of the digital rural construction level and the green transformation efficiency of cultivated land utilization are significantly positive in 2011–2020, indicating the significant spatial autocorrelation between the two. Therefore, it is sensible to conduct research by using spatial econometric models.

### 5.2. Regression Analysis of Spatial Dubin Model

As shown in Table 9, the regression coefficient of the digital rural construction level is positive, passing the test at the 1% statistical level. It is demonstrated that the higher the level of digital rural construction in the province, the greater the efficiency of green transformation of cultivated land use in the province. In the meantime, the regression coefficient of the spatial lag term of the digital rural construction level is also positive, passing the test under the significance level of 1%. That is to say, the level of digital rural construction produces a significant spatial effect, and the level of digital rural construction in this province also plays a significant positive role in the green transformation efficiency of cultivated land use for neighboring provinces. Given the inability of the regression coefficient of the SDM model to reflect the direct effect of the independent variable on the dependent variable and the actual spatial spillover effect, the partial differential method proposed by Le Sage and Pace [64] is used in this study to divide the impact coefficient of the digital rural construction level on the efficiency of green transformation of cultivated land use into direct effect, indirect effect and total effect. Among them, the direct effect is defined as the impact of the level of digital village construction in the province on the efficiency of green transformation of cultivated land use in the province, and the indirect effect is referred to as the impact of the level of digital village construction in the province on the efficiency of green transformation of cultivated land use in neighboring provinces, as reflected in the spatial spillover effect of the level of digital village construction. The total effect is defined as the sum of direct effect and indirect effect. Table 9 shows the decomposition results of spatial effects. The direct effects, indirect effects and total effects of the digital rural construction level all pass the 1% significance test and have positive values. It suggests that the level of digital rural construction in this province not only improves the green transformation efficiency of cultivated land use in this province, but also enhances the green transformation efficiency of cultivated land use in neighboring provinces. Thus, the hypothesis H2 proposed in this study is supported.

### 5.3. Digital Rural Construction and Green Transformation of Regional Cultivated Land Utilization

According to the above analysis, there is a spatial spillover effect caused by the impact of digital rural construction on the efficiency of green transformation of cultivated land use. With the constant improvement of digital infrastructure and the further development of digital technology, regional information communication and economic exchange become increasingly convenient and faster. Digital technology can be applied to break the limits of time and space, promote the cross regional flow of various production factors such as capital, labor and advanced production technology, and optimize the allocation of resources among different regions. By strengthening the construction of the digital countryside, not only can the efficiency of green transformation of cultivated land use be improved in this region, but the efficiency of green transformation of cultivated land use can also be enhanced in surrounding areas through the spillover of production factors. This leads to the improvement of efficiency in the green transformation of cultivated land use in a wider range. Digital technology can improve the efficiency of green transformation of cultivated land utilization, which to some extent reduces the contradiction between the enhancement of cultivated l and utilization intensity and environmental protection. Through the scale effect of green transformation of regional cultivated land utilization, regional ecological protection can be promoted and the scale economy of regional cultivated land utilization can be strengthened.

## 6. Conclusions

In this study, the impact mechanism of digital rural construction on the green transformation efficiency of cultivated land use is described in detail for the comprehensive measurement of China’s inter provincial digital rural construction level and green transformation efficiency of cultivated land utilization from 2011 to 2020. On this basis, the promoting effect of digital rural construction on green transformation efficiency of cultivated land rate is empirically tested. Furthermore, a discussion is conducted around the spatial spillover effect of digital rural construction through the spatial Dubin model. The conclusions of this study are as follows. Firstly, digital rural construction plays a positive role in the efficiency of regional cultivated land use green transformation. This result remains valid after various robustness tests are carried out, such as the replacement model, the elimination of municipal samples, the lag of independent variables and endogenous treatment. Secondly, the digital rural construction shows significant regional heterogeneity and distribution heterogeneity in improving the efficiency of green transformation for regional cultivated land use. This promoting effect is more significant in the eastern region than in the central and western regions. As the efficiency of green transformation of cultivated land utilization improves, the promoting effect of digital village shows an increasing trend in terms of marginal utility. Thirdly, the level of digital rural construction and the efficiency of green transformation of cultivated land use show a significant positive spatial autocorrelation. Apart from improving the green transformation efficiency of cultivated land use in this region, digital rural construction also enhances the green transformation efficiency of cultivated land use in the surrounding regions. That is to say, the promotion of digital rural construction produces a positive spatial spillover effect. Based on the above research conclusions, the following policy recommendations are made.

Firstly, the process of digital rural construction should be accelerated. By increasing investment in the access, operation and maintenance of digital technology facilities in rural areas, the construction of digital villages can be effectively promoted. It is necessary to promote the comprehensive and in-depth integration and application of new generation digital information technology and agricultural production and operation. Through a precise agricultural production mode, the construction of digital countryside can provide a crucial driving force for the transformation of green cultivated land use. Secondly, it is worth paying attention to the differences in the process of digital rural construction. Due to the level of economic development, geographical conditions and other factors, there is a significant heterogeneity in the level of digital rural construction across China. The “digital gap” between different regions can be reduced by promoting the construction of digital villages in the eastern developed regions and increasing support for the construction of digital villages in the less developed regions in the central and western regions. Thus, the construction of digital villages can promote the transformation of green use of cultivated land in an all-round way. Finally, a regional cooperation mechanism should be established by preserving the “welfare sharing” characteristics of digital rural construction. Digital rural construction can not only enhance the efficiency of green transformation of cultivated land use in this region, but also produces a positive spatial spillover effect. The internet and other digital media should be taken advantage of to promote the full flow of agricultural green production concepts. New technologies and high-quality talents reinforce the resultant force of agricultural green development across regions and promote the coordinated level of green cultivated land utilization in various provinces.

In this study, there are certain limitations. Firstly, the evaluation system for the green transformation efficiency of cultivated land use is not as comprehensive as required. As a complex process, the green transformation of cultivated land use system involves not only the green transformation of material elements, but also the green transformation of non-material elements such as the production concept and labor quality of farmers. However, due to the availability of data, this paper excludes the non-material elements in the evaluation index system. Therefore, they can be included in the evaluation index system in the future to better reflect the connotation of green transformation of cultivated land use. Secondly, due to the space limit, this paper is limited to analyzing the spatial spillover effect of digital rural construction on the green transformation efficiency of cultivated land use from a macro perspective. As a result, the influence of social and economic factors in different regions is ignored. This spatial spillover effect may vary as well. In order to obtain more practical results, this spatial spillover effect can be tested from the perspective of regional heterogeneity in the future.

## Figures and Tables

**Table 1:** Evaluation index system of green transformation efficiency of cultivated land use.

Variable	Variable	Index	Index Meaning
Input	Land	The sown area of crops per worker	Total planting area of crops/number of employees in planting industry (hm^2^/People)
	Labor force	Number of employees in planting industry	Number of employees in planting industry (10,000)
		Number of agricultural technicians in state-owned enterprises and institutions	Number of agricultural technicians in state-owned enterprises and institutions (person)
	Technology	Comprehensive utilization rate of crops	Weighted average value of machine tillage rate, machine seeding rate and machine yield (%)
	Capital	Fertilizer input	Amount of chemical fertilizer converted into pure fertilizer (10,000 t)
		Pesticide input	Pesticide usage (10,000 t)
		Input of agricultural film	Amount of agricultural film used (10,000 t)
		Irrigation input area	Effective irrigation area (thousand hm^2^)
		Diesel input	Diesel consumption (10,000 t)
Expected output	Economic effect	Agricultural output value	Output value of planting industry (100 million yuan)
	Social effect	grain yield	Grain output (10,000 t)
Unexpected output	Agricultural carbon emissions	Total carbon emissions from cultivated land use	Total agricultural carbon emissions (10,000 t)
	Agricultural non-point source pollution	Agricultural non-point source pollution emissions	Total agricultural non-point source pollution (10,000 t)

**Table 2:** Digital rural construction level evaluation index system.

Variables	Sub Level Variables	Weight
Infrastructure construction of digital village	Average computer ownership per 100 rural households	0.17147
	Average number of mobile phones owned by rural households per 100 households	0.07637
	Rural broadband access users	0.43865
Service level of digital village	Total digital inclusive finance Index	0.12209
	Length of rural delivery route	0.19142

**Table 3:** Descriptive Statistics.

Variables	Number of Samples	Mean	Std. Dev.	Min	Max
Green transformation efficiency of cultivated land use (GTCLU)	300	0.295	0.145	0.037	0.751
Digital rural construction level (Dig)	300	0.309	0.161	0.042	0.832
Affected area (Dis)	300	6.313	1.709	0	8.838
Rural medical facility level (Med)	300	10.488	3.684	3.997	20.489
Planting structure (Plant)	300	0.649	0.140	0.355	0.971
Multiple crop index (Multi)	300	1.294	0.395	0.486	2.341

**Table 4:** Benchmark regression results.

Variables	POLS	OLS—FE1	OLS—FE2
Dig	0.204 *** (0.065)	0.162 *** (0. 048)	0.215 *** (0.055)
Dis			−0. 114 *** (0.013)
Med			0.016 ** (0.008)
Plant			0.155 (0.242)
Multi			0.133 *** (0.042)
Constant	0.955 *** (0.026)	1.037 *** (0.022)	0.719 *** (0.175)
Province	NO	YES	YES
Year	NO	YES	YES
R^2^	0.513	0.641	0.754
OBS	300	300	300

Note: ** and *** are significant at 5% and 1% levels respectively, and the standard error values are in brackets.

**Table 5:** Robustness Test.

Variables	Tobit Model	Municipalities Are Excluded from Samples	Lagging One Period of Core Explanatory Variables	Tool Variable Method
Dig	0.216 *** (0.036)	0.208 *** (0.044)		0.307 *** (0.099)
L. Dig			0.217 *** (0.053)	
Dis	−0.114 *** (0.017)	−0.157 *** (0.030)	−0.163 *** (0.012)	−0.192 *** (0.028)
Med	0.016 ** (0.008)	0.009 * (0.005)	0.011 *** (0.005)	0.008 ** (0.004)
Plant	0.153 (0.214)	0.175 (0.183)	0.177 (0.283)	0.200 (0.375)
Multi	0.130 *** (0.013)	0.187 *** (0.052)	0.149 *** (0.045)	0.135 *** (0.029)
Constant	0.719 *** (0.188)	0.833 *** (0.267)	0.763 *** (0.252)	0.884 *** (0.391)
Province	YES	YES	YES	YES
Particular year	YES	YES	YES	YES
Weak identification F test				65.410 ***
Endogenous test *p*				0.004
R^2^		0.742	0.750	0.685
OBS	300	260	270	300

Note: *, ** and *** are significant at 10%, 5% and 1% levels respectively, and the standard error values are in brackets.

**Table 6:** Results of regional heterogeneity.

Variables	The Eastern Region	The Central Region	The Western Region
Dig	0.241 *** (0.031)	0.147 *** (0.052)	0.177 *** (0.043)
Dis	−0.089 *** (0.008)	−0.075 ** (0.006)	−0.103 *** (0.002)
Med	0.027 *** (0.014)	0.014 ** (0.007)	0.008 ** (0.004)
Plant	0.186 (0.287)	1.025 (1.412)	0.068 * (0.040)
Multi	0.067 *** (0.028)	0.104 *** (0.031)	0.142 *** (0.039)
Constant	0.840 *** (0.329)	0.619 *** (0.235)	0.868 *** (0.305)
Provinces	YES	YES	YES
Year	YES	YES	YES
R^2^	0.735	0.587	0.694
OBS	110	80	110

Note: *, **, *** are significant at 10%, 5% and 1% levels respectively, and the standard error values are in brackets. The eastern region includes Beijing, Tianjin, Hebei, Liaoning, Shanghai, Jiangsu, Zhejiang, Fujian, Shandong, Guangdong and Hainan; the central region includes Shanxi, Jilin, Heilongjiang, Anhui, Jiangxi, Henan, Hubei and Hunan; the western region includes Inner Mongolia, Guangxi, Chongqing, Sichuan, Guizhou, Yunnan, Shaanxi, Gansu, Qinghai, Ningxia and Xinjiang.

**Table 7:** Distribution Heterogeneity Results.

Variables	25%	50%	75%	90%
Dig	0.200 *** (0.027)	0.241 *** (0.038)	0.268 *** (0.060)	0.316 *** (0.109)
Dis	−0.078 *** (0.008)	−0.117 *** (0.014)	−0.160 *** (0.051)	−0.216 *** (0.074)
Med	0.009 * (0.006)	0.012 * (0.007)	0.120 *** (0.018)	0.146 *** (0.022)
Plant	0.124 (0.179)	0.287 (0.235)	0.437 (0.368)	0.469 (0.387)
Multi	0.116 *** (0.014)	0.163 *** (0.024)	0.198 *** (0.054)	1.002 *** (0.032)
Constant	0.433 ** (0.210)	0.382 *** (0.122)	0.305 *** (0.110)	0.473 *** (0.137)
Provinces	YES	YES	YES	YES
Year	YES	YES	YES	YES
OBS	300	300	300	300

Note: *, **, *** are significant at 10%, 5% and 1% levels respectively, and the standard error values are in brackets.

**Table 8:** Spatial autocorrelation test.

Variables	2011	2012	2013	2014	2015	2016	2017	2018	2019	2020
Dig	0.333 ***	0.379 ***	0.389 ***	0.380 ***	0.376 ***	0.298 ***	0.274 ***	0.258 ***	0.256 ***	0.235 ***
GTCLU	0.193 ***	0.292 ***	0.339 ***	0.298 ***	0.171 ***	0.391 ***	0.302 ***	0.380 ***	0.407 ***	0.416 ***

Note: *** means significant at 1% level.

**Table 9:** Regression results of spatial Dubin model.

	SDM	Direct Effect	Indirect Effect	Total Effect
Dig	0.163 *** (0.014)	0.192 *** (0.021)	0.240 *** (0.062)	0.432 *** (0.075)
Dis	−0.088 *** (0.007)	−0.126 *** (0.021)	−0.194 (0.137)	−0.320 *** (0.047)
Med	0.007 ** (0.004)	0.008 ** (0.004)	0.011 (0.009)	0.019 ** (0.008)
Plant	0.116 (0.126)	0.164 (0.118)	0.290 (0.167)	0.454 (0.402)
Multi	0.104 ** (0.051)	0.143 *** (0.030)	0.086 * (0.044)	0.229 *** (0.013)
W * Dig	0.227 *** (0.042)			
W * Dis	−0.147 (0.093)			
W * Med	0.015 (0.009)			
W * Plant	−0.178 (1.002)			
W * Multi	0.073 ** (0.032)			
Spatial rho	0.260 ***			

Note: *, **, *** are significant at 10%, 5% and 1% levels respectively, and the standard error values are in brackets.

## Data Availability

The sample data are sourced from the corresponding years of “China Statistical Yearbook”, “China Rural Statistical Yearbook”, “China Agricultural Machinery Industry Yearbook” and “China Science and Technology Statistical Yearbook” and the report on digital inclusive finance index compiled by the Digital Finance Research Center of Peking University.
